# *%svy_logistic_regression*: A generic SAS macro for simple and multiple logistic regression and creating quality publication-ready tables using survey or non-survey data

**DOI:** 10.1371/journal.pone.0214262

**Published:** 2019-09-03

**Authors:** Jacques Muthusi, Samuel Mwalili, Peter Young

**Affiliations:** Division of Global HIV and Tuberculosis, U.S. Centers for Disease Control and Prevention, Nairobi, Kenya; University College London, UNITED KINGDOM

## Abstract

**Introduction:**

Reproducible research is increasingly gaining interest in the research community. Automating the production of research manuscript tables from statistical software can help increase the reproducibility of findings. Logistic regression is used in studying disease prevalence and associated factors in epidemiological studies and can be easily performed using widely available software including SAS, SUDAAN, Stata or R. However, output from these software must be processed further to make it readily presentable. There exists a number of procedures developed to organize regression output, though many of them suffer limitations of flexibility, complexity, lack of validation checks for input parameters, as well as inability to incorporate survey design.

**Methods:**

We developed a SAS macro, ***%svy_logistic_regression***, for fitting simple and multiple logistic regression models. The macro also creates quality publication-ready tables using survey or non-survey data which aims to increase transparency of data analyses. It further significantly reduces turn-around time for conducting analysis and preparing output tables while also addressing the limitations of existing procedures. In addition, the macro allows for user-specific actions to handle missing data as well as use of replication-based variance estimation methods.

**Results:**

We demonstrate the use of the macro in the analysis of the 2013–2014 National Health and Nutrition Examination Survey (NHANES), a complex survey designed to assess the health and nutritional status of adults and children in the United States. The output presented here is directly from the macro and is consistent with how regression results are often presented in the epidemiological and biomedical literature, with unadjusted and adjusted model results presented side by side.

**Conclusions:**

The SAS code presented in this macro is comprehensive, easy to follow, manipulate and to extend to other areas of interest. It can also be incorporated quickly by the statistician for immediate use. It is an especially valuable tool for generating quality, easy to review tables which can be incorporated directly in a publication.

## Introduction

The principles of reproducible research are increasingly gaining interest both in the research community [1–5] and in the popular imagination because of high-profile failures to reproduce results. While funders and journals are increasingly requiring both publications and their supporting data be made publicly available with few exceptions [6–8] there has been less focus on the reproducibility of the analysis process itself. Reproducible research refers to increasing the transparency of the research endeavor by making the initial data, detailed analysis steps, and tools available to allow others to reproduce ones’ findings. Peng and Leek refer to increasing reproducibility as a tool to reduce the time required to uncover errors in analysis [9]. One important link in the reproducible research value chain is eliminating manual reformatting of results from statistical software into draft manuscript tables.

In most epidemiological studies, one of the main outcomes of interest is disease prevalence–i.e. the proportion of all study subjects with a disease. Researchers are often interested in the probability or odds of subjects having a disease as well as associated predictive factors. These factors can be categorical (such as gender), ordinal (for example age categories), or continuous (for instance duration on treatment). The measures of association are often presented as crude (unadjusted) odds ratios from simple logistic regression or they can be presented as adjusted odds ratios from multiple logistic regression. In scientific reports from observational epidemiological studies it is common to combine the results from multiple statistical models and present the odds ratios side by side in complex tables showing the association between multiple covariates with the outcome of interest, both unadjusted and adjusted.

Logistic regression models can be fitted easily using available standard statistical analysis software such as SAS, SUDAAN, Stata or R, among others, and have been extended to handle weights and/or specialized variance estimation to account for complex survey designs. However, output from these software is not formatted for use directly in a publication and must be re-organized in order to make it more presentable based on the cultural norms of the biomedical literature or the specific requirements of the scientific journal [10, 11]. Most epidemiological publications present regression tables showing odds ratios estimates and the corresponding 95% confidence intervals and/or p-values. They further enrich the output by including frequencies and proportion of study subjects who experienced the outcome of interest. Results from simple and multiple regression can also be presented side-by-side in one table. Some examples of publications that adopt this convention of presenting regression results are provided in the references [12–15]. In order to accomplish this, one has to manually copy different parts of output into a template. This is both time-consuming and potentially prone to errors when revisions to the analysis are required.

A number of programs have been developed to facilitate conversion of regression output from statistical analysis software into formatted tables for publications. In Stata, several programs including ***esttab*** [16, 17], ***reformat*** [18], ***outreg2*** [19] are useful in formatting regression output. In R such packages as ***stargazer*** [20], ***broom*** [21], ***flextable*** [22] have also being found helpful. Though they are useful to statisticians, they suffer from numerous limitations. For instance, they cannot automatically combine results from several simple logistic regression into a single table. It is also not possible to combine results from simple and multiple logistic regression into one output table. They are also not fully generic in that one has to explicitly specify variable labels and levels of categorical variables instead of extracting these from metadata. Further manipulation of output, for example, concatenating odds ratios and the corresponding 95% confidence interval into one column cell, has to be done manually which increases the risk of typographical errors in the output table. In SAS software, logistic regression models can be fitted using the LOGISTIC, GENMOD and SURVEYLOGISTIC procedures [23], though output from these procedures must be formatted further to make it presentable. SAS provides a flexible and powerful macro language that can be utilized to create and populate numerous table templates for presenting regression results. However, limited programming work has being done in SAS to date. There are several SAS macros including ***%table1*** [24], ***%logistic_table*** [25] and ***%UniLogistic*** [26] which have been developed to assist in processing the output from regression procedures, but they are largely limited in terms of flexibility, lack of support for complex survey designs, or are unable to incorporate both categorical and continuous variables in one macro call. For instance, the macro, ***%table1*,** presents variable names instead of the more meaningful variable labels. The other macros, ***%logistic_table*,** and ***%UniLogistic*,** produce output from simple logistic regression but not from multiple logistic regression. Also the ***%UniLogistic*** macro does not accommodate survey design parameters. Furthermore, these macros lack validation checks for input parameters and also do not export the output into word processing and spreadsheet programs for ease of incorporating into a publication.

## Methods

### Sample survey methods

Sample surveys permit description of a population using a sample rather than studying the entire population. The sample can be selected using various methods. Commonly used approaches are simple random sampling, stratified sampling, clustered sampling, and multi-stage sampling. In simple random sampling, each unit of the population has equal probability of being selected. It is often used as a benchmark for comparison with other methods [27]. Stratification involves partitioning the population into subgroups according to the levels of a stratification variable, so that the outcome variable is more homogenous within each stratum than in the population as a whole. The stratifying variable is usually a key population unit characteristic such as sex, age, residency or geographic region and should be known before the sampling process begins. Stratification is usually done to increase precision as well as to obtain inferences about the strata [27–29]. In multi-stage sampling, the sample is selected in a hierarchical approach starting with a primary sampling unit (PSU) within which secondary sampling units (SSU) are selected within which tertiary sampling units (TSU) may be selected and so forth. For example, in a survey we can select a school as the primary sampling unit within which classes are selected in the second stage. Pupils are then selected in the third stage with the selected schools. Multi-stage design facilitates fieldwork. Clustering refers to the fact several non-independent units, clusters, are selected simultaneously [27]. To ensure proper representation, sample selection probabilities from the survey design method are computed. The corresponding survey/sampling weights are then computed as the inverse of selection probability [27, 28, 30].

### The standard logistic regression model

Consider a binary response variable *Y*, which takes one or two possible values denoted by 1 or 0. For example, *Y* = 1 if a disease is present, otherwise *Y* = 0). Let ***X*** = (*X*_1_,*X*_2_,…,*X*_*p*_) be a vector of independent variables and *π*(*x*) = Pr(*Y* = 1|***X*** = *x*) is the probability of response to be modelled (where *π*(*x*) ranges between 0 and 1) as a function of ***X***. The binary response follows a Bernoulli distribution and the corresponding logistic regression model takes the form:

$$\ln\left(\frac{\pi (x)}{1-\pi (x)}\right)=\alpha +\beta^{\prime }X$$ (1)

where *α* is the intercept and ***β*** = (*β*_1_,*β*_2_,…,*β*_*s*_)′ is the vector of ***s*** slopes. The predicted probability of the response variable is denoted by

$$\pi (x)=\frac{\exp\left(\alpha +\beta^{\prime }X\right)}{1+\exp\left(\alpha +\beta^{\prime }X\right)}$$ (2)

The odds of response = 1 is given by

$$\frac{\pi (x)}{1-\pi (x)}=\exp\left(\alpha +\beta^{\prime }X\right)$$ (3)

Both *α* and ***β*** parameters are estimated by the maximum likelihood estimation (MLE) method. Under MLE, it is assumed that observations are independent and identically distributed. Details of MLE method have been discussed in detail in [31, 32].

### Logistic regression model with sample survey data

Complex survey designs combine two or more sampling designs to form a composite sampling design. The observations selected under complex surveys are no longer independent; hence, the standard logistic regression model is inappropriate in this context. The general computation method for a logistic regression model with complex survey design is demonstrated as follows:

Let *U* = {1,2,…,*N*} be a finite population, divided into *h* = 1,2,…,*H* strata. Each stratum is again divided into *j* = 1,2,…,*n*_*h*_ PSU each of which is made up of *i* = 1,2,…,*n*_*hj*_ SSU corresponding to *n*_*hji*_ units. Let the observed data consist of $n_{hj}^{\prime }$ SSU chosen from $n_{h}^{\prime }$ PSU in stratum *h*. The total number of observations is then given by $n=\sum_{h=1}^{H}\sum_{j=1}^{n_{h}^{\prime }}\sum_{i=1}^{n_{hj}^{\prime }}n_{hji}$. The *hjik*−th sampling unit has an associated sampling weight, which is equal to the inverse of its sampling probability, denoted by $w_{hjik}=\frac{1}{\pi_{hjik}}$. Let *Y*_*hjik*_ denote the binary response outcome variable that takes the values 1 or 0, ***x***_*hjik*_ is the covariate matrix and ***β*** denotes the regression coefficients. The logistic regression model for a stratified clustered multi-stage sampling design is given by:

$$logit\left(\pi (x_{hjik})\right)=\ln\left(\frac{\pi (x_{hjik})}{1-\pi (x_{hjik})}\right)=x_{hjik}^{\prime }\beta$$ (4)

and the predicted probability of the response variable is denoted by:

$$\pi (x_{hjik})=\frac{\exp\left(x_{hjik}^{\prime }\beta \right)}{1+\exp\left(x_{hjik}^{\prime }\beta \right)}$$ (5)

while the odds of response = 1 is given by

$$\frac{\pi (x_{hijk})}{1-\pi (x_{hijk})}=\exp\left(x_{hjik}^{\prime }\beta \right)$$ (6)

The ***β*** are then estimated using the maximum pseudo-likelihood method that incorporates the sampling design and the different sampling weights. References [32–35] present detailed description of the maximum pseudo-likelihood method.

### The *%svy_logistic_regression* SAS macro

Recognizing the limitations of existing tools, we have designed a SAS macro, ***%svy_logistic_regression***, to help overcome these shortcomings while supporting the principles of reproducible research. The macro specifically organizes output from SAS procedures and formats it into quality publication epidemiologic tables containing regression results. We describe the macro functionality, provide an example analysis of a publicly available dataset, and provide access to the source code for the macro to allow others to use and extend it to support their own reproducible research.

Our developed SAS macro allows for both simple and multiple logistic regression analysis. Moreover, this SAS macro combines the results from simple and multiple logistic regression analysis into a single quality publication-ready table. The layout of the resulting table is consistent with how models are often presented in the epidemiological and biomedical literature, with unadjusted and adjusted model results presented side by side.

The macro, written in SAS software version 9.3 [36], runs logistic regression analysis in a sequential and interactive manner starting with simple logistic regression models followed by multiple logistic regression models using SAS PROC SURVEYLOGISTIC procedure. Frequencies and totals are obtained using PROC SURVEYMEANS and PROC SURVEYFREQ procedures. The final output is then processed using PROC TEMPLATE, PROC REPORT procedures and the output delivery system (ODS).

The macro is made up of six sub-macros. The first sub-macro, ***%svy_unilogit***, fine-tunes the dataset by applying the conditional statements, and computing the analysis domain size, thus preparing a final analysis dataset. It also prepares the class variables and associated reference categories. It calls the second and third sub-macros, ***%svy_logitc*** and ***%svy_logitn***, to perform separate simple (survey) logistic regression model on each categorical or continuous predictor variable respectively. It further processes results outputs into one table. The fourth sub-macro, ***%svy_multilogit***, performs multiple (survey) logistic regression on selected categorical and continuous predictor variables and processes result outputs into one table. The fifth sub-macro, ***%svy_printlogit***, combines results from ***%svy_unilogit*** and ***%svy_multilogit*** sub-macros and processes the output into an easy to review table which is exported into Microsoft word processing and excel spreadsheet programs. In addition, where survey design variables have been specified the macro automatically incorporates them into the computation. The sixth sub-macro, ***%runquit***, is executed after each SAS procedure or DATA step, to enforce in-build SAS validation checks on the input parameters. These include but not limited to checking if the specified dataset exists, ensuring required variables are specified, and verifying that values for reference categories for outcome, domain and categorical variables exist and are valid, as well as checking for logical errors. Once an error is encountered, the macro stops further execution and prints the error message on the log for the user to address it.

The macro is generic in that it can be used to analyze any dataset intended to fit a logistic regression model from survey or non-survey settings. It accepts both categorical and continuous predictor variables. Where survey data are used, it allows one to specify design-specific variables such as strata, clusters or weights. Domain analysis for sub-population estimation is also provided for by the macro. Ignoring domain analysis and instead performing a subset analysis will lead to incorrect standard errors. For non-survey settings, the survey input parameters like weights and cluster are set to a default value of 1.

The macro also allows the user to explicitly specify the level or category of the binary outcome variable to model as well as reference categories for categorical predictor variables. Further, it runs sequentially by first producing results from simple logistic regression from which the user can select predictor variables to include into the multiple logistic regression, then combine the results of multiple models into a single table. Apart from including only significant predictor variables, based on global/type3 p-values, the user can also choose to include any other variables deemed important by subject matter experts. This flexibility allows for specification of such variables as confounders or effect modifiers even when they are not statistically significant in the simple logistic model. It further provides the user with an opportunity to identify variables with unstable estimates and/or are highly variable during execution of ***%svy_unilogit*** sub-macro. The user can then exclude these variables when they run the ***%svy_multilogit*** sub-macro. The final output is then processed into a quality publication-ready table and exported into word processing and spreadsheet programs for use in the publication, or if needed, for further hand editing by the authors.

The user must provide input parameters as specified in Table 1. Unless stated (optional), the other parameters must be provided so that the macro can execute successfully. The ***outevent*** parameter and reference categories for class variables are case sensitive and must be specified in the case they appear in the data dictionary. All other parameters are mainly dataset variables and may be specified in any case. We use lower case for this demonstration. Validation checks enforce these requirements, simplifying debugging errors in macro invocation. The statistician only interacts with sub-macros 1, 4 and 5 by providing input parameters. If a permanent SAS dataset is to be analyzed, the LIBNAME statement can be used to indicate the path or folder where the dataset is located.

**Table 1:** Input parameters for *%svy_unilogit*, *%svy_multilogit* and *%svy_printlogit* macros.

Parameter	Description
***%svy_unilogit*** and ***%svy_multilogit*** macros	
dataset	name of input dataset
outcome	name of dependent binary variable of interest e.g., hiv_status
outevent	value label of outcome variable (without quotation) to model e.g., Positive, in the case of modeling Hepatitis A risk factors
catvars	list of categorical explanatory variables (nominal or ordinal) separated by space e.g., age category (in years) which takes the categories; 1 = “20–39”, 2 = “40–59”, 3 = “> = 60”
class	class statement for categorical explanatory variables specifying the baseline (reference) category e.g., age_category (ref = “> = 60”)
contvars	list of continuous explanatory variables separated by space e.g., Age (in years) which takes values from 20 to 70 years
condition	(optional) any conditional statements to create and or fine-tune the final analysis dataset specified using one IF statement
strata	(optional) survey stratification variable
cluster	(optional) survey clustering variable
weight	(optional) survey weighting variable
domain	(optional) domain variable for sub-population analysis
missval_lab	(optional) value label for missing values. If missing data have a format, it should be provided, otherwise macro assumes the default format “.”
varmethod	(optional) value for variance estimation method namely Taylor (the default) or replication-based variance estimation methods including JK or BRR
rep_weights_values	(optional) values for REPWEIGHTS statement, but may be specified with replication-based variance estimation method is JK or BRR
varmethod_opts	(optional) options for variance estimation method, e.g., jkcoef = 1 df = 25 for JK
missval_opts	(optional) options for handling missing data within proc survey statement, e.g., “MISSING” or “NOMCAR”. If no option is specified missing observations are excluded from the analysis
print	variable for displaying/suppressing the output table on the output window which takes the values (NO = suppress output, YES = show output)
***%svysvy_printlogit*** macro	
tablename	short name of output table
tabletitle	title of output table
outcome & outevent	same as defined in ***%svy_unilogit*** and ***%svy_multilogit*** macros
outdir	directory for saving output files
***%runquit*** macro	
syserr	SAS in-build macro variable that checks presence of any system errors

If missing values are present in the data, SAS SURVEYLOGISTIC procedure by default excludes these from analysis based on the assumption that the missing values are missing completely at random (MCAR). In some cases, missing values may not be missing completely at random and the NOMCAR option can be specified to include these in variance estimation. The MISSING option may also be used in cases where survey design variables (cluster, strata, or domain) have missing values so that they may not be excluded from the analysis. These options are passed to the macro using the missval_opts parameter.

By default, SAS SURVEYLOGISTIC procedure uses Taylor series method to estimate variance. If data for replication-based methods such as Jackknife (JK) or Balanced Repeated Replication (BRR) are available, one can specify these methods in the macro using the _varmethod parameter. Where JK or BRR methods are specified, users may also specify values for REPWEIGHTS statement using the _rep_weights_values parameter. To customize the replication process, users can add SURVEYLOGISTIC procedure options using the varmethod_opts parameter. [23, 37, 38] provides detailed description of the use of replication methods for variance estimating in sample surveys.

Because of the flexible nature of the macro, users are provided with an opportunity to identify variables with unstable estimates and/or very wide 95% CI when they run the simple logistic regression implemented by ***%svy_unilogit*** sub-macro. The user should then exclude these variables when they run the multiple logistic regression model implemented by the ***%svy_multilogit*** sub-macro to avoid model convergence problems.

## Results

### Example: Analysis of NHANES dataset

We demonstrate the use of our macro in the analysis of the 2013–2014 National Health and Nutrition Examination Survey (NHANES), a suite of complex surveys designed to assess the health and nutritional status of adults and children in the United States (U.S.). In brief, the main objectives of the survey were to estimate and monitor trends in prevalence of selected diseases, risk behaviors and environmental exposures among targeted populations, to explore emerging public health issues, and to provide baseline health characteristics for other administrative use [39].

NHANES used a four-stage, stratified sampling design, where counties were selected as primary sampling units (PSUs) using probability proportionate to size (PPS) in the first stage. The second stage involved selecting sections of counties that consisted of a block containing a cluster of households with approximately equal sample sizes per PSU. Dwelling units including households were then selected in the third stage with approximately equal selection probabilities. Individuals within a household were selected in the fourth stage. Stratification was done based on selected demographics characteristics of PSUs. Survey weights were then computed using the various sampling probabilities to account for the complex survey design. The data files are freely available to the public on the NHANES website at: https://www.cdc.gov/nchs/nhanes/Index.htm. See [40] for more details regarding the NHANES survey design and contents.

**Fig 1:**
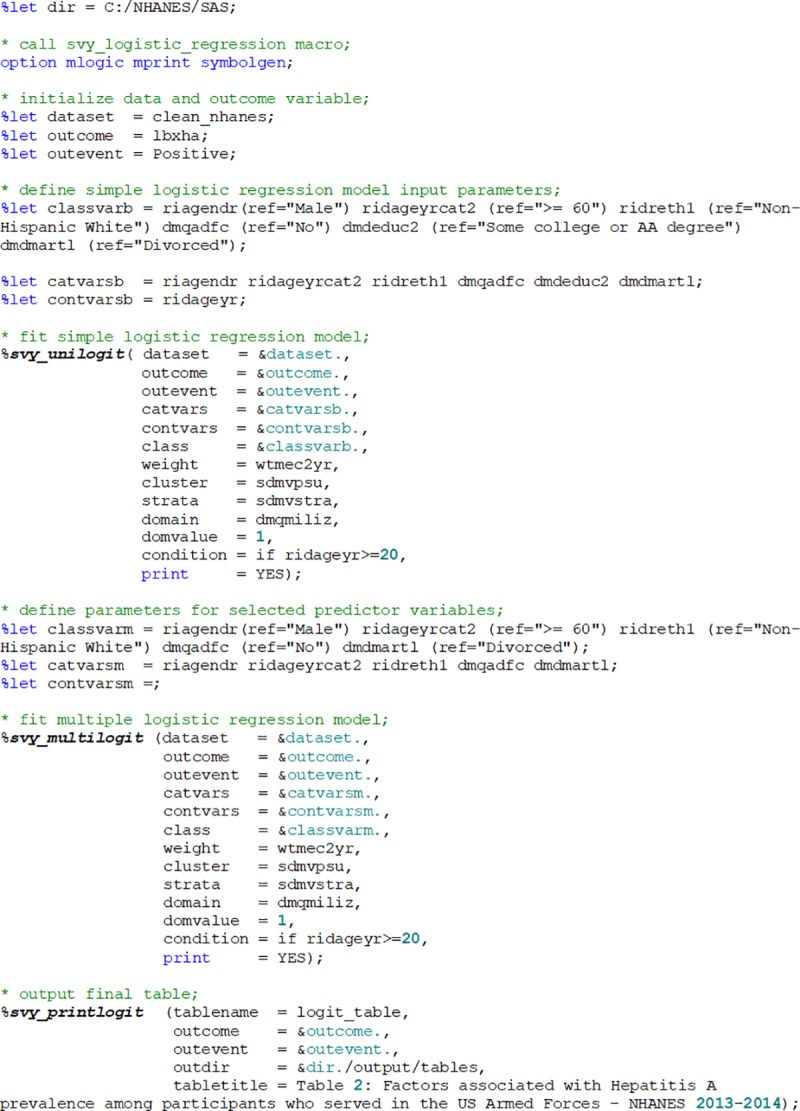
Sample *%svy_logistic_regression* macro call.

The dataset (clean_nhanes) used in this example includes participants’ socio-demographic characteristics including riagendr (Gender), ridageyr (Age in years at screening), ridreth1, (Race/Hispanic origin), dmqadfc (Service in a foreign country), dmdeduc2 (Education level among adults aged 20+ years), and dmdmartl (Marital status). The binary outcome variable is lbxha (Hepatitis A antibody test result). The aim of the analysis is to investigate factors associated with a positive test for Hepatitis A antibody among participants aged 20+ years who have served active duty in the U.S. Armed Forces (dmqmiliz). Appropriate survey weights wtmec2yr (sample weights for participants with a medical examination) were applied. The macros were run with user-defined parameters. The user should explicitly specify the reference category for factor variables and for the binary outcome, as shown in Fig 1.

The complete SAS output consists of several tables, the majority of which are auxiliary and are used to help in processing the output. Two important output tables are the simple and the multiple logistic regression tables. The simple logistic regression table shows result of bivariate regression as shown in Table 2.

**Table 2:** Output of simple logistic regression model results from *%svy_unilogit* macro.

Factor	N^@^	Freq^&^	OR_CI^$^	p_value^α^	g_p_value^β^
Gender					
Male	473	205 (37.7)	ref		
Female	35	15 (39.7)	1.09 (0.40–2.92)	0.857	0.857
Total	508	220 (37.9)			
Age category in years at screening					
> = 60	322	131 (30.7)	ref		
20–39	51	39 (83.0)	11.0 (5.10–23.7)	< .001	< .001
40–59	135	50 (31.2)	1.02 (0.58–1.79)	0.934	
Total	508	220 (37.9)			
Race/Hispanic origin					
Non-Hispanic White	307	114 (34.1)	ref		
Mexican American	23	14 (67.1)	3.93 (1.14–13.5)	0.018	< .001
Non-Hispanic Black	126	59 (46.1)	1.65 (1.12–2.43)	0.006	
Other Hispanic	26	17 (64.3)	3.47 (0.92–13.1)	0.046	
Other Race	26	16 (52.3)	2.12 (0.60–7.53)	0.207	
Total	508	220 (37.9)			
Served in a foreign country					
No	243	86 (27.4)	ref		
Yes	264	134 (48.6)	2.51 (1.32–4.77)	0.002	0.002
Total	507	220 (38.1)			
Education level					
College graduate or above	147	51 (32.0)	ref		
9-11th grade	37	16 (33.4)	1.07 (0.50–2.28)	0.857	0.296
High school graduate	122	56 (41.1)	1.48 (0.83–2.65)	0.147	
Less than 9th grade	9	5 (42.6)	1.58 (0.42–6.02)	0.465	
Some college or AA degree	193	92 (41.8)	1.53 (0.90–2.61)	0.089	
Total	508	220 (37.9)			
Marital status					
Separated	11	5 (27.3)	ref		
Divorced	75	30 (29.4)	1.11 (0.36–3.42)	0.847	0.029
Living with partner	17	9 (60.2)	4.02 (0.81–19.8)	0.063	
Married	311	130 (36.3)	1.52 (0.53–4.37)	0.403	
Never married	48	21 (51.4)	2.81 (0.72–10.9)	0.104	
Widowed	46	25 (44.0)	2.09 (0.66–6.62)	0.175	
Total	508	220 (37.9)			
Age in years at screening	508	220 (37.9)	0.97 (0.95–0.98)	< .001	< .001

^@^ = Total number of observations

^&^ = Frequency of prevalent cases (and weighted prevalence in percentage)

^$^ = Weighted Odds Ratio (95% confidence interval)

^α^ = Class level p-value

^β^ = Global/Type 3 p-value

The table consists of six variables, namely: Factor (risk factor variable), N (total frequency of observations), Freq (frequency of prevalent cases and corresponding weighted prevalence in percentage), OR_CI (weighted odds ratio and 95% confidence interval) p_value (class level p-value), g_p_value (global/type3 p-value). Typically the analyst/researcher selects statistically-important risk factors based on the global/type3 p-values. From this example, all risk factors except gender and education level were statistically significant. However, based on epidemiological considerations, gender and age are often treated as potential confounder variables. Thus they are included in the multiple logistic regression model regardless of statistical significance. Another important aspect to pick from Table 2 is the frequency columns which show the sample size for each factor and each level of the factor. In this example the expected total measurements for each factor was n = 508 out of which 220 (37.8%) tested positive for Hepatitis A antibody. All other factors except service in a foreign country (n = 507) had complete information available. The importance of this is to ensure that factors with substantive proportion of complete information are selected for inclusion in the multiple logistic regression model. In addition, the row percentages provide guidance on the choice of reference category of factor variables. However, for ordinal factors it is often advisable to use the lowest or highest category as reference, depending on the outcome of interest. After selecting all important variables, the ***%svy_multilogit*** macro is then executed. The ***%svy_printlogit*** macro automatically processes the output into a quality easy to review output as shown in Table 3.

**Table 3:** Quality publication-ready output from the *%svy_printlogit* macro combining results from *%svy_unilogit* and *%svy_multilogit* macros.

	Hepatitis A antibody ^€^		Unadjusted odds ratios			Adjusted odds ratios		
Characteristic	Total^¥^	Positive^£^ n (%)^&^	OR ^ξ^ (95% CI) ^$^	p-value^α^	Type3^β^ p-value	OR (95% CI)	p-value	Type3 p-value
Gender								
Male	473	205 (37.7)	ref					
Female	35	15 (39.7)	1.09 (0.40–2.92)	0.857	0.857	1.00 (0.24–4.15)	0.998	0.998
Total	508	220 (37.9)						
Age category in years at screening								
> = 60	322	131 (30.7)	ref					
20–39	51	39 (83.0)	11.0 (5.10–23.7)	< .001	< .001	13.8 (5.15–36.7)	< .001	< .001
40–59	135	50 (31.2)	1.02 (0.58–1.79)	0.934		1.27 (0.54–2.99)	0.544	
Total	508	220 (37.9)						
Race/Hispanic origin								
Non-Hispanic White	307	114 (34.1)	ref					
Mexican American	23	14 (67.1)	3.93 (1.14–13.5)	0.018	< .001	3.41 (1.01–11.5)	0.032	0.01
Non-Hispanic Black	126	59 (46.1)	1.65 (1.12–2.43)	0.006		1.91 (1.08–3.37)	0.016	
Other Hispanic	26	17 (64.3)	3.47 (0.92–13.1)	0.046		3.40 (0.53–21.6)	0.159	
Other Race	26	16 (52.3)	2.12 (0.60–7.53)	0.207		1.42 (0.28–7.28)	0.647	
Total	508	220 (37.9)						
Served in a foreign country								
No	243	86 (27.4)	ref					
Yes	264	134 (48.6)	2.51 (1.32–4.77)	0.002	0.002	3.09 (1.85–5.17)	< .001	< .001
Total	507	220 (38.1)						
Education level								
College graduate or above	147	51 (32.0)	ref					
9-11th grade	37	16 (33.4)	1.07 (0.50–2.28)	0.857	0.296			
High school graduate	122	56 (41.1)	1.48 (0.83–2.65)	0.147				
Less than 9th grade	9	5 (42.6)	1.58 (0.42–6.02)	0.465				
Some college or AA degree	193	92 (41.8)	1.53 (0.90–2.61)	0.089				
Total	508	220 (37.9)						
Marital status								
Separated	11	5 (27.3)	ref					
Divorced	75	30 (29.4)	1.11 (0.36–3.42)	0.847	0.029	1.09 (0.31–3.81)	0.884	0.017
Living with partner	17	9 (60.2)	4.02 (0.81–19.8)	0.063		1.96 (0.42–9.05)	0.349	
Married	311	130 (36.3)	1.52 (0.53–4.37)	0.403		1.69 (0.55–5.14)	0.318	
Never married	48	21 (51.4)	2.81 (0.72–10.9)	0.104		1.67 (0.47–5.97)	0.387	
Widowed	46	25 (44.0)	2.09 (0.66–6.62)	0.175		3.45 (0.89–13.4)	0.051	
Total	508	220 (37.9)						
Age in years at screening	508	220 (37.9)	0.97 (0.95–0.98)	< .001	< .001			

^¥^ = Total number of observations

^€^ = Outcome variable label

^£^ = Outcome value label of category of interest

^&^ = Frequency of prevalent cases (and weighted prevalence in percentages)

^ξ^ = Weighted Odds Ratio

^$^ = Weighted 95% confidence interval for Odds Ratio

^α^ = Class level p-value

^β^ = Global/Type3 p-value

## Discussion

This paper presents an elegant and flexible SAS macro, ***%svy_logistic_regression***, for producing quality publication-ready tables from unadjusted and adjusted logistic regression analyses. Even though a number of SAS macros are available on the internet for processing output from logistic regression into a publication-ready table, they are complex to follow and/or have limited features, thus restricting their adoption. Many macros are not generic and hence can only be used with the data for which they were designed.

The SAS macro presented here is generalized, highly suitable to handle different scenarios, and is simple to implement and invoke from user macros. In addition, our macro includes the row or column total and frequency of prevalent cases of each variable level, which can immediately allow the analyst/researcher to identify levels with sparse data. Row percentages help the researcher in the choice of reference category. Global or type3 p-values shows whether a variable is an important predictor. Individual p-values shows if a given variable category is comparable to the reference category. The macro provides validation checks on the input parameters including the dataset, variables and values of variables to ensure that the analyst obtains valid estimates. The output of this SAS macro helps improve efficiency of knowledge generation by reducing the steps required from analysis to clear and concise presentation of results. The macros importantly supports replication-based JK and BRR, which are now gaining use in complex survey data analysis.

## Conclusion

As our contribution to the emerging field of reproducible research, we have provided source code for the SAS macro as well as expected outputs using a publicly available dataset. By publishing this macro, it will allow other SAS macro programmers and users to verify and build upon this code. Production of publication-quality tables is increasingly important as data analyses become more complex, involving larger datasets and requiring more sophisticated computations and tabulation, notwithstanding the need for quick results. This macro helps to make data analysis results readily available, and allows one to publish data summaries in a single document, thus allowing others to easily execute the same code to obtain the same results. The quality, publication-ready results from this macro are suitable for direct inclusion in manuscripts for peer-reviewed journals. The macro can also be used to routinely generate standardized tables. This is especially useful for disease surveillance systems where the same analyses are repeated on a quarterly or annual basis. We hope the published results from this macro will provide information and inspiration for further research.

## Supporting information

S1 Code The SAS macro *% svy_logistic_regression* source code. The source code for the SAS macro to perform univariate and multivariate logistic regression analyses and a simple example of the implementation.
(TXT)

S2 Code Sample code for *% svy_logistic_regression* macro call. A sample SAS code to read in the data and call the macro. The **S1 Code** and **S2 Code** are also available online at https://github.com/kmuthusi/generic-sas-macros and have been labelled as “%*svy_logistic_regression*.*sas*” and “*svy logistic regression anafile*.*sas*” respectively.
(TXT)

S1 Data Sample NHANES dataset used to demonstrate the functioning of the manuscript. The complete NHANES dataset is freely available to the public on the NHANES website at: https://www.cdc.gov/nchs/nhanes/Index.htm.
(ZIP)

## References

[1] Peng RD, Dominici F, Zeger SL. Reproducible epidemiologic research. American Journal of Epidemiology. 2006;163(9):783–9. 10.1093/aje/kwj093 . 16510544

[2] Peng RD. Reproducible research in computational science. Science. 2011;334(6060):1226–7. 10.1126/science.1213847 22144613 PMC3383002

[3] Peng RD. Reproducible research and Biostatistics. Biostatistics. 2009;10(3):405–8. 10.1093/biostatistics/kxp014 . 19535325

[4] Iqbal SA, Wallach JD, Khoury MJ, Schully SD, Ioannidis JP. Reproducible Research Practices and Transparency across the Biomedical Literature. PLoS Biol. 2016;14(1):e1002333 10.1371/journal.pbio.1002333 26726926 PMC4699702

[5] Arnold Tim, Kuhfeld Warren F. Using SAS and LATEX to Create Documents with Reproducible Results. URL: http://supportsascom/resources/papers/proceedings12/324-2012pdf. 2012.

[6] Wellcome Trust. Policy on data, software and materials management and sharing 2017 [January 16, 2018].

[7] Wellcome Trust. Open access policy 2017 [January 16, 2018].

[8] U S Department of Health and Human Services. Open Government Plan. 2016. p. 48–9.

[9] Leek JT, Peng RD. Opinion: Reproducible research can still be wrong: adopting a prevention approach. Proc Natl Acad Sci U S A. 2015;112(6):1645–6. 10.1073/pnas.1421412111 25670866 PMC4330755

[10] Altman DG, Gore SM, Gardner MJ, Pocock SJ. Statistical guidelines for contributors to medical journals. British Medical Journal (Clinical research ed). 1983;286(6376):1489–93. 6405856 10.1136/bmj.286.6376.1489 PMC1547706

[11] Lang TA, Altman DG. Basic statistical reporting for articles published in clinical medical journals: the SAMPL Guidelines In: Smart, Maisonneuve, Polderman A (eds). Science Editors' Handbook, European Association of Science Editors 2013.

[12] Nala R, Cummings B, Horth R, Inguane C, Benedetti M, Chissano M, et al Men who have sex with men in Mozambique: identifying a hidden population at high-risk for HIV. AIDS Behavior. 2015;19(2):393–404. 10.1007/s10461-014-0895-8 25234252 PMC4341016

[13] Moore DM, Cui Z, Lachowsky N, Raymond HF, Roth E, Rich A, et al HIV Community Viral Load and Factors Associated With Elevated Viremia Among a Community-Based Sample of Men Who Have Sex With Men in Vancouver, Canada. Journal of Acquired Immune Deficiency Syndrome. 2016;72(1):87–95. 10.1097/QAI.0000000000000934 26825177 PMC4837069

[14] Cherutich P, Kim AA, Kellogg TA, Sherr K, Waruru A, De Cock KM, et al Detectable HIV Viral Load in Kenya: Data from a Population-Based Survey. PLoS One. 2016;11(5):e0154318 10.1371/journal.pone.0154318 27192052 PMC4871583

[15] Oluoch T, Katana A, Kwaro D, Santas X, Langat P, Mwalili S, et al Effect of a clinical decision support system on early action on immunological treatment failure in patients with HIV in Kenya: a cluster randomised controlled trial. The Lancet HIV. 2016;3(2):e76–e84. 10.1016/S2352-3018(15)00242-8 26847229 PMC4777604

[16] Jann B. Making regression tables from stored estimates. The Stata Journal, URL: http://wwwstata-journalcom/articlehtml?article=st0085. 2005;5(3):288–308.

[17] Jann B. Making regression tables simplified. The Stata Journal, URL: http://wwwstata-journalcom/articlehtml?article=st0085_1. 2007;7(2):227–44.

[18] Brady T. REFORMAT: Stata module to reformat regression output. Statistical Software Components S426304, Boston College Department of Economics, revised 06 Oct 2002, URL: https://ideasrepecorg/c/boc/bocode/s426304html. 2002.

[19] Wada R. OUTREG2: Stata module to arrange regression outputs into an illustrative table. Statistical Software Components S456416, Boston College Department of Economics, revised 17 Aug 2014 URL: https://ideasrepecorg/c/boc/bocode/s456416html. 2005.

[20] Hlavac M. stargazer: Well-Formatted Regression and Summary Statistics Tables. R package version 5.2.1. https://CRAN.R-project.org/package=stargazer. 2018.

[21] Robinson D, Gomez M, Demeshev B, Menne D, Nutter B, Luke J, et al. broom: Convert Statistical Analysis Objects into Tidy Data Frames. URL: https://cran.r-project.org/package=broom. 2017.

[22] Gohel D, Nazarov M. flextable: Functions for Tabular Reporting. URL: https://cran.r-project.org/package=flextable. 2018.

[23] SAS Institute Inc. SAS/STAT 9.3 User’s Guide. Cary, NC: SAS Institute Inc; 2011.

[24] Gravely A, Clothier B, Nugent S. Creating an Easy to Use, Dynamic, Flexible Summary Table Macro with P-Values in SAS for Research Studies. Proceedings from MidWest SAS Users Group Paper AA072014.

[25] Qi J. Automating the Process of Generating Publication Quality Regression Tables through SAS Base Programming. Proceedings from MiwWest SAS Users Group Paper BB232016.

[26] Dhand NK. UniLogistic: A SAS Macro for Descriptive and Univariable Logistic Regression Analyses. Journal of Statistical Software. 2010;35(1):1–15.21603108

[27] Levy PS, Lemeshow S. Sampling of Populations: Methods and Applications. 4th ed. New York: John Wiley & Sons; 2013.

[28] Cochran WG. Sampling Techniques. 3rd Edition ed. New York: John Wiley & Sons; 1977.

[29] Foreman EK. Survey Sampling Principles. New York: Marcel Dekker; 1991.

[30] Kish L. Survey Sampling. New York: John Wiley & Sons 1965.

[31] Agresti A. Categorical Data Analysis. Second ed. New York: John Wiley & Sons; 2002.

[32] Hosmer DW, Lemeshow S. Applied Logistic Regression. Second ed. New York: John Wiley & Sons; 2000.

[33] Lee ES, Forthofer RN. Analyzing Complex Survey Data. Second ed. Thousand Oaks: Sage; 2006.

[34] Archer KJ, Lemeshow S, Hosmer DW. Goodness-of-Fit Tests for Logistic Regression Models When Data Are Collected Using a Complex Sampling Design. Computational Statistics and Data Analysis. 2007;51:4450–64. 10.1016/j.csda.2006.07.006.

[35] Lumley T. Analysis of Complex Survey Samples. Journal of Statistical Software. 2004;9:1–19. 10.18637/jss.v009.i08.

[36] SAS Institute Inc. Base SAS 9.3. Cary, NC: SAS Institute Inc; 2011.

[37] Lohr SL. Sampling: Design and Analysis. 2nd edition ed. Boston: Brooks/Cole; 2010.

[38] Wolter KM. Introduction to Variance Estimation. New York: Springer-Verlag; 1985.

[39] Centers for Disease Control and Prevention. Centers for Disease Control and Prevention (CDC). National Center for Health Statistics (NCHS). National Health and Nutrition Examination Survey Data. Hyattsville, MD: U.S. Department of Health and Human Services, Centers for Disease Control and Prevention, 2013–2014, URL: https://www.cdc.gov/nchs/nhanes/Index.htm: National Center for Health Statistics (NCHS); 2015.

[40] Johnson CL, Dohrmann SM, Burt VL, Mohadjer LK. National Health and Nutrition Examination Survey: Sample design, 2011–2014. National Center for Health Statistics. Vital and Health Statistics. 2014;2 (162). 25569458

